# MAITabolism^2^ – the emerging understanding of MAIT cell metabolism and their role in metabolic disease

**DOI:** 10.3389/fimmu.2022.1108071

**Published:** 2023-01-19

**Authors:** Nidhi Kedia-Mehta, Andrew E. Hogan

**Affiliations:** ^1^ Kathleen Lonsdale Institute for Human Health Research, Maynooth University, Maynooth, Co Kildare, Ireland; ^2^ Obesity Immunology Group, Education and Research Centre, St Vincent’s University Hospital, University College Dublin, Dublin, Ireland; ^3^ National Children’s Research Centre, Dublin, Ireland

**Keywords:** MAIT, immunometabolism, metabolic disease, obesity, diabetes

## Abstract

Mucosal associated invariant T (MAIT) cells are a population of unconventional innate T cells due to their non-MHC restriction and rapid effector responses. MAIT cells can recognise bacterial derived antigens presented on the MHC-like protein *via* their semi-restricted T cell receptor (TCR). Upon TCR triggering MAIT cells rapidly produce a range of effector molecules including cytokines, lytic granules and chemokines. This rapid and robust effector response makes MAIT cells critical in host responses against many bacterial pathogens. MAIT cells can also respond independent of their TCR *via* innate cytokines such as interleukin (IL)-18, triggering cytokine production, and are important in anti-viral responses. In addition to their protective role, MAIT cells have been implicated in numerous inflammatory diseases, including metabolic diseases often contributing to the pathogenesis *via* their robust cytokine production. Effector cells such as MAIT cells require significant amounts of energy to support their potent responses, and the type of nutrients available can dictate the functionality of the cell. Although data on MAIT cell metabolism is just emerging, several recent studies are starting to define the intrinsic metabolic requirements and regulators of MAIT cells. In this review we will outline our current understanding of MAIT cell metabolism, and outline their role in metabolic disease, and how disease-related changes in extrinsic metabolism can alter MAIT cell responses.

## Introduction

Metabolism can be defined as the process of breaking down fuel (e.g., glucose or lipids) to generate energy or biosynthetic molecules (e.g., proteins or lipids). These processes can be further divided in those that happen at a whole-body level (systemic metabolism) and those that happen within individual cells (cellular metabolism). Both are critically important for normal function, with alterations in either resulting in the potential development of pathophysiology. Mucosal associated invariant T (MAIT) cells are a population of unconventional T cells which have emerged as a critically important player in the human immune system (1). They can protect the host from invading pathogens ranging from bacterium and fungi to viruses, and are capable of killing malignant cells (2). However, dysregulated MAIT cells can drive the development of chronic diseases such as cancer and metabolic disease (3–5). In this review, we will summarize our emerging understanding of the cellular metabolic pathways regulating MAIT cells, and their role as major contributors to the development of metabolic diseases like obesity and diabetes.

## MAIT cell basics

MAIT cells are a population of unconventional T cells found across numerous human tissues (2, 6). These cells are unconventional in that they are restricted by MR1 opposed to MHC molecules (6). They also express a very limited T cell receptor (TCR) repertoire defined by the invariant TCR alpha chain Vα7.2 (6), which usually pairs with limited Vβ chains with Vβ2 or Vβ13 being amongst the most prevalent. MAIT cells recognize bacterial derived metabolites presented on the MHC like molecule MR1, which also supports their development (7, 8). In humans MAIT cells are relatively abundant, representing approximately 2-8% of peripheral blood T cells, 3-10% of adipose tissue T cells and up to 50% of hepatic T cells, of note, MAIT cell frequencies are significantly lower in mice (2). The majority of MAIT cells are CD8+, but subsets of CD4+ and double negative (CD8-/CD4-) have been reported (9, 10). Other defining phenotypic markers of MAIT cells include the c-type lectin CD161 and the IL-18 receptor (1). MAIT cells express several key transcription factors which define their functional capabilities including TBET, RORγt and the innate master transcription factor PLZF.

MAIT cells can be activated in two distinct manners, TCR dependent activation *via* MR1 presentation of microbial antigens or TCR independent activation *via* cytokines such as IL-18 (7, 11). Upon activation, MAIT cells can rapidly produce a wide range of cytokines and lytic molecules including interferon (IFN)γ, interleukin(IL)-17 and granzyme B, which supports MAIT cell host defensive responses (12–15). Several studies have demonstrated that MAIT cells can lyse both bacterially infected cells and transformed malignant cells in an MR1 dependent manner (14–16). MAIT cells can also be activated independent of their TCR *via* innate cytokines, the best described being IL-12, IL-18 and type 1 interferons (11, 17–20). During viral infection these cytokines can act synergistically to activate MAIT cells, driving robust production of IFNγ and granzyme B, however there is little evidence for MR1-independent cytotoxicity. *In vivo* studies support the concept that both TCR dependent and independent activation synergize to drive optimal MAIT cell responses. Most published studies have highlighted host protection as the primary function of MAIT cells. However, emerging data provides compelling evidence that MAIT cells also play an important role in tissue homeostasis and repair (21–23).

## Cellular immunometabolism

Cellular metabolism has emerged to be an important process that supports and regulates immune cell function (24). Apart from being a source of ATP production, metabolism also sustains macromolecule synthesis, intracellular signaling and post translational modifications. All these cellular pathways support immune cell effector function and proliferation. Immune cells adopt different cellular metabolic pathways depending on their functional needs (25). For instance, naïve T cells are quiescent and rely on oxidative phosphorylation for the maintenance, while activated T cells that are highly proliferative relying on glycolytic metabolism (26). This switch to glycolysis may seem redundant in terms of energy production but a higher flux through the glycolytic pathway supports biosynthesis as well as sustaining high energy demands (27). The enzyme GAPDH has been shown to directly influence immune function in CD4 T cells, through post translational control of the IFNγ mRNA transcription (28).

Metabolic reprogramming in T and natural killer (NK) cells is controlled by the nutrient sensor mTORC1 (29–31)and the transcription factor MYC (32, 33). In the absence of mTORC1 and MYC, T cells and NK cells fail to upregulate glycolysis and effector functions (32, 34–38). Both mTORC1 and MYC are highly sensitive to nutrient availability in the cellular microenvironment (32, 39). Therefore, cellular metabolism is major regulator of immune function and is fine-tuned by optimal nutrient availability in the microenvironment.

## Cellular metabolism and MAIT cells

**Figure 1:**
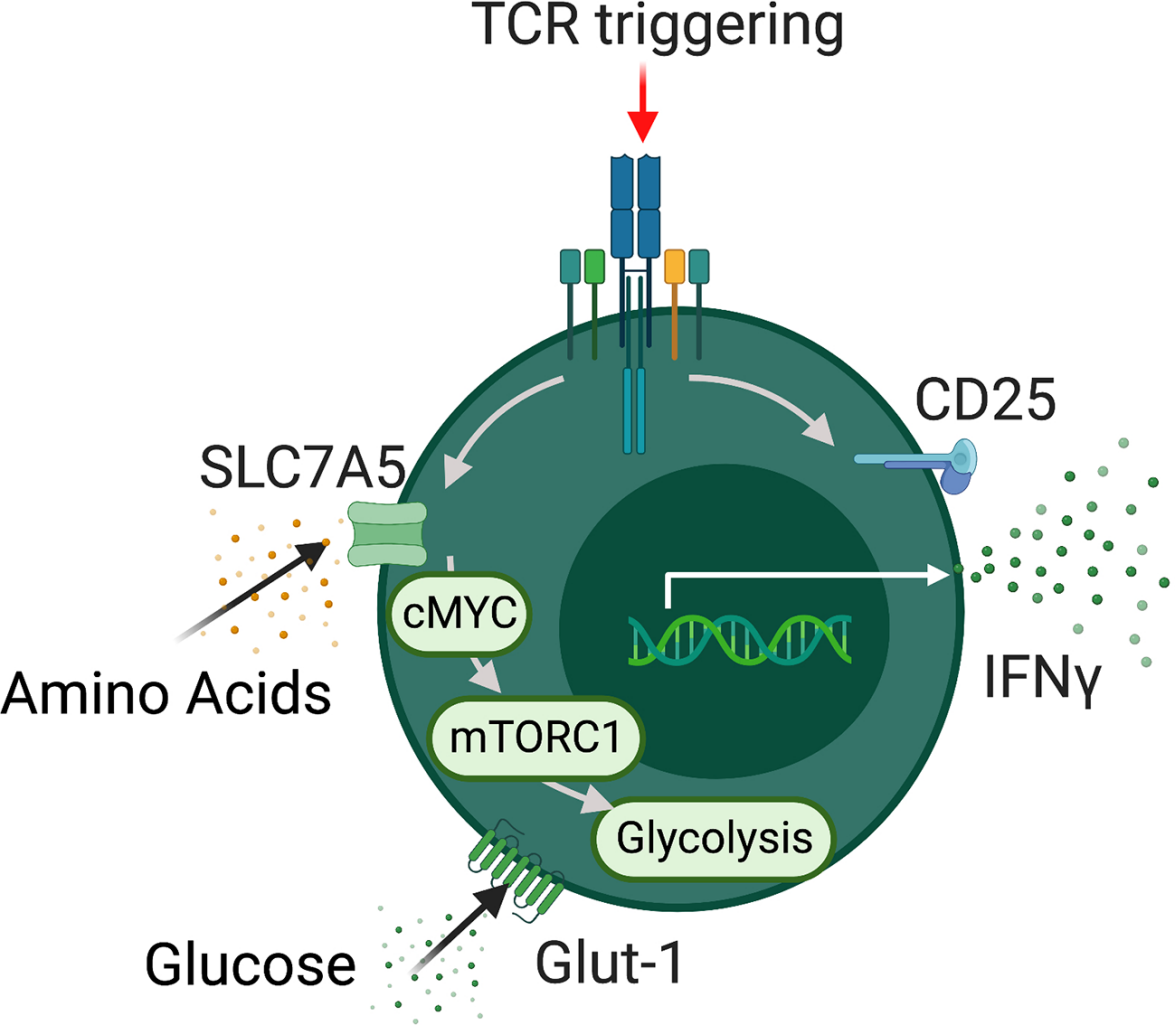
MAIT cells metabolism. Schematic detailing metabolic programming in TCR activated MAIT cells. Activated MAIT cells upregulate a range of nutrient transporters including SLC7A5 and GLUT-1 followed increased rates of glycolysis which support effector function. In addition MAIT cell metabolic programming is dependent on the metabolic regulators mTORC1 and MYC.

The intrinsic metabolic pathways and regulators utilized by MAIT cells are rapidly emerging with several studies published in the past three years (40–43) (**Figure 1**). These studies have focused on the metabolic control of specific MAIT cell functions, such as cytokine production or proliferation. We will discuss the metabolic pathways used by MAIT in the context of individual functions. Upon activation, MAIT cells rapidly spring into action, growing, dividing, and synthesizing new effector molecules, these processes require significant energy and supply of biointermediates. Our research group has recently highlighted MYC as a critical metabolic regulator, central to MAIT cell growth and proliferation (43). MYC is a well-established metabolic master regulator in conventional T cells, controlling the expression of nutrient transporters and downstream metabolic pathways (37, 38). MYC deficient T cells demonstrate reduced growth proliferation and effector function (37). In MAIT cells, MYC is rapidly activated after stimulation, leading to the expression of several surface receptors essential for MAIT cell growth and proliferation, including IL-2 receptor alpha and CD98, each of which is essential for MAIT cell proliferation (43). Downstream of MYC, activated MAIT cells increase their rates of glycolysis, metabolizing glucose, which supports their growth and proliferation.

In 2019, our group also highlighted the importance of glycolytic metabolism for MAIT cell production of IFNγ (41). Inhibition of glycolysis using 2-deoxy-glucose severely limited MAIT cell production of IFNγ, but interestingly has no significant effect of TNF. Limiting the substrate for glycolysis, glucose, also restricted IFNγ production by MAIT cells (41). These findings were in agreement with studies demonstrating the critical importance of glycolysis metabolism for effector T cell production of IFNγ (40). In addition to cytokines such as IFNγ, MAIT cells are also prolific producers of lytic molecules such as granzyme B. In 2019, the Klenerman group elucidated the metabolic pathways required for granzyme B production by MAIT cells (40). In line with our findings, they found that MAIT cell rapidly elevate their rates of glycolysis upon activation, and like IFNγ this was essential for their production of granzyme B (40). Whilst trying to unravel the metabolic pathways regulating MAIT cell effector molecular production, an unusual anomaly arose, MAIT cells in healthy human produce very little IL-17 but display high rates of glycolytic metabolism. In other IL-17 producing T cell populations, such as Th17 cells, glycolysis supported the IL-17 programme (44, 45). In patients with obesity, the frequencies of IL-17 producing MAIT cells are expanded (46, 47), but glycolytic metabolism is defective suggesting glycolysis may not support IL-17 production by MAIT cells (41). In support of this concept, we found that MAIT cells from people with obesity had altered mitochondria, with depolarized membrane potential and elevated levels of reactive oxygen species (ROS) (42). In conventional T cells, mitochondrial ROS (mROS) has been linked to elevated IL-17 (48), and we demonstrated that reducing mROS levels in obese MAIT cells limited IL-17 production, linking IL-17 to mitochondrial metabolism. In a recent pre-print from the Kronenberg lab, single cell sequencing of murine MAIT cells subsets revealed elevated uptake of fatty acids, lipid content, and mitochondrial potential in IL-17 producing MAIT cells when compared to IFNγ producing MAIT cell subsets (49).

With cellular metabolism having such a major influence on MAIT cell biology and effector function, it is very likely that altered microenvironments (e.g. limited glucose in tumour microenvironments) in disease settings may change or dysregulate MAIT cell metabolism, ultimately shifting them towards the dysfunctional responses reported in many diseases. One group of diseases where large shifts in the microenvironment (e.g. altered nutrients and elevated inflammation) are most prevalent are the metabolic diseases. In the remainder of this mini-review we will outline the available literature on the impact of metabolic disease on MAIT cells.

## Systemic metabolism and MAIT cells

Since their discovery and characterization, MAIT cells have been investigated in numerous chronic disease settings, from multiple sclerosis to psoriasis to several types of connective tissue diseases, often implicated in pathogenesis (50–55). One of the most investigated disease clusters has been the metabolic diseases; with studies in obesity, diabetes (both type 1 and 2), non-alcoholic fatty liver disease and cardiovascular disease (46, 47, 56–59). In the remainder of this review, we will discuss the published studies on MAIT cells in the various metabolic diseases (**Table 1**).

**Table 1:** Table outlining the phenotype, frequency and role of MAIT cells in various metabolic diseaes.

Metabolic Disease	Cytokine IFN TNF IL17 IL4	Activation Markers	Circulating MAIT cell #	Cellular Metabolism	Role	Additional Data	Refs
Steady State	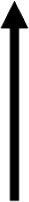 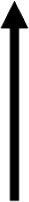	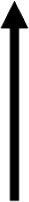	N/A	Glycolysis MYC & mTOR	Protective	N/A	40–43
Obesity	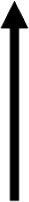	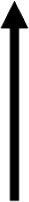	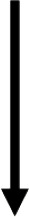	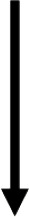	Pathogenic	Exhausted with high ROS	41–43, 46, 47, 56
T1DM	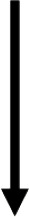 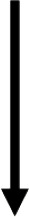	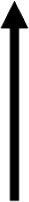 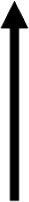	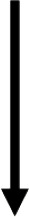	Unknown	Pathogenic	Different in recent onset vs established	56, 59–62
T2DM	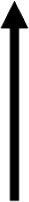	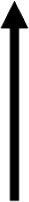 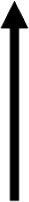	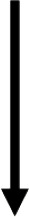	Unknown	Pathogenic	Linked to gut dysbiosis	46, 63, 64
NAFLD	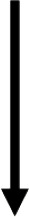 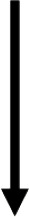 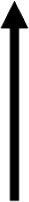	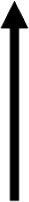 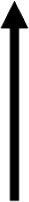	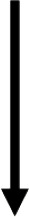	Unknown	Protective/ Pathogenic	Polarization of macrophages may protect but can cause fibrosis	57, 65, 66
CVD	Not Measured	Not Measured	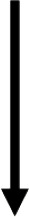	Unknown	Unknown	N/A	58, 67

Upwards arrow means the corresponding cytokine above is elevated & downward means reduced.

## Obesity

Obesity is a chronic progressive disease in which excess adiposity impairs health and metabolic homeostasis (68). Underpinning obesity related ill-health is significant immune dysregulation and inflammation, which can start early in life (69, 70). The first published studies detailing MAIT cells in metabolic disease focused on their frequencies, cytokine profile and adipose tissue residency in adults with obesity. In two independent studies MAIT cell frequencies in peripheral blood where reduced compared to healthy controls, and it was demonstrated that MAIT cells accumulate in human adipose tissue with reduced numbers in people with obesity (46, 47). In addition, both studies demonstrated altered cytokine profiles with elevated IL-17 in both peripheral blood and adipose tissue, suggesting a potential pathogenic role. In a subsequent study, O’Brien et al. also demonstrated elevated IL-17 producing MAIT cells in people with obesity, and as outlined earlier the elevated IL-17 was linked to elevated mitochondrial ROS, which could be reduced with the addition of ROS scavengers (42).

Building on the observational studies in humans, Toubal and colleagues provided the strongest data to date linking MAIT cells to the development of metabolic dysregulation using murine models of obesity (56). Mice deficient in MAIT cells (due to genetic deletion of MR1) were protected from the metabolic complications of diet induced obesity. This finding was further validated by the exacerbation of metabolic complications in transgenic mice with human levels of MAIT cells (56). Again, it was demonstrated that IL-17 producing MAIT cells were expanded in the setting of obesity. Recently our group aimed to elucidate a mechanistic link between IL-17 producing MAIT cells and the development of obesity related metabolic dysregulation. In a cohort of children with obesity, we noted a strong correlation between IL-17 producing MAIT cells and HOMA-IR, a measure of insulin resistance (63). To investigate if IL-17 producing MAIT cells could directly disrupt insulin mediated glucose uptake we cultured human skeletal muscle cells in the presence of the MAIT cell secretome from either healthy controls or people with obesity. We observed reduced insulin mediated glucose uptake in the presence of “obese” MAIT cell secretome. The addition of an IL-17 neutralizing antibody, partially reversed the impact of the obese MAIT cell secretome, highlighting IL-17 as a causative factor, and further supporting the concept of MAIT cells as a pathogenic player in obesity and the development of metabolic diseases such as type 2 diabetes mellitus.

## Type 2 diabetes mellitus

One of the most prevalent metabolic diseases associated with obesity is Type 2 Diabetes Mellitus (T2DM). In the original study from Magalhaes and colleagues, their cohort of patients with obesity was further stratified into patients with or without T2DM (46). Circulating MAIT cell frequency was further reduced in people with T2DM (46). In a study by Zhang and colleagues they showed activation induced cell death of MAIT cells was amplified in patients with T2DM, maybe explaining the reports of reduced frequencies (64). This increased cell death was associated with increased expression of OX40 (CD134) on MAIT cells from patients with T2DM. In addition to altered frequencies, MAIT cells from patients with T2DM also displayed a heightened IL-17 type cytokine profile (46). Of note, increased expression of CD134 is linked to IL-17 production in other IL-17 producing T cells (71). In murine models of obesity, MAIT cells increased gut leakiness and promoted gut microbiota dysbiosis in obesity (56). Several studies have linked gut microbiota dysbiosis to the pathogenesis of T2DM (72–75).

## Metabolic associated fatty liver disease

Metabolic or Non-Alcoholic Associated Fatty Liver Disease (NAFLD) is another condition associated with high fat diet and metabolic disharmony, characterized by the excess accumulation of fat in the liver (76). While MAIT cells were found to contribute to the pathogenesis of T2DM, their role in NAFLD is less clear. In one study, circulating MAIT cells in patients with NAFLD were more activated and expressed the liver homing receptor CXCR6. These MAIT cells could promote M2 polarization *in vitro* in an IL4 dependent manner. In the mouse model of NAFLD, MAIT cells induced Th2 phenotype. Moreover, mice lacking MAIT cells promoted steatohepatitis and induced pro-inflammatory cytokines with increased TNF and resulted in M1 macrophage polarization (65). However, in a separate study, it was demonstrated that MAIT cells could also contribute to pathogenicity of cirrhosis by promoting liver fibrosis (57). A recent study found that patients with NAFLD treated for weight loss for 12 weeks either by diet intervention (DI) or exercise intervention (EI) showed differential outcomes and independent benefits with respect to MAIT cells. In patients following DI, there was a reduction in the activation marker CD69 on MAIT cells and an improvement in histological steatosis grade whereas in patients who followed exercise intervention, there was an increase in the apoptotic marker CD95 in circulating as well as intrahepatic MAIT cells with a reduction in frequencies of intrahepatic MAIT cells alongside improvements in fibrosis and ballooning seen in NAFLD (66).

## Type 1 diabetes mellitus

Type 1 Diabetes Mellitus (T1DM) is an autoimmune disease, characterized by destruction of insulin producing β cells of Langerhans (77). This condition is mediated by an inappropriate activation of both innate and adaptive immune systems resulting in the occurrence of anti-islet autoantibodies and autoreactive T cells (77). MAIT cells are dysregulated in children with recent onset T1DM, with reduced frequencies (59, 60). An increase in migratory receptors could also indicate increased homing of MAIT cells toward inflamed tissues. In addition to altered frequencies and phenotype, MAIT cells from children with recent-onset T1DM produced less IFNγ than those from control children, whereas their production of TNF, IL-4 and Granzyme B was higher. Like children with T1DM, alterations in MAIT cell were observed in adults with long term T1D compared to adults who had a recent onset of T1DM. Long-term T1DM showed increased CD25 expression and decreased levels of IFNγ and TNF in adults compared with recent onset T1DM. These MAIT cells alterations exacerbated in an autoimmune context in females. Females that had another associated autoimmune condition had an elevated CD69 expression and a decreased BCL2 expression compared with females without an autoimmune condition indicating an activated and exhausted phenotype in chronically affected study participants (61). Building on their observations in children with T1DM, Rouxel and colleagues demonstrated exacerbated diabetes in MAIT cell deficient NOD mice suggesting a protective role for MAIT cells (59). This effect was associated with the involvement of MAIT cells in maintaining gut integrity which in turn regulates DC activation. Excessive DC activation could contribute to development of T1DM by priming anti islet auto-reactive T cells (59). However, in the same study, MAIT cells were found to infiltrate the pancreas of NOD mice where they could potentially contribute to pathology by producing IFNγ and GrB (59). In a follow up study from Kuric et al, they did not find an influx of MAIT cells into the pancreas of patients with T1DM, however it should be noted the sample size was limited, and MAIT cell were detected using IHC and PCR as opposed to flow cytometric analysis using MR1 tetramers like in the Rouxel study. Therefore, further experiments in human are required to determine if MAIT cells are accumulating in the pancreas of patients with T1DM (62).

## Cardiovascular diseases

Cardiovascular diseases which include coronary heart disease, hypertension and stroke combined are the leading cause of death and are more frequent in individuals with obesity (78). Studies investigating MAIT cells in the context of cardiometabolic diseases are limited. In one study, Touch et al. found that MAIT cell numbers were reduced in cardio metabolic disorders such as coronary artery disease (CAD) and congestive heart failure (CHF), with a more profound loss of MAIT cell frequencies in (CAD-CHF) (58). The research findings showed that total T cells numbers did not change irrespective of the disease state. The decreased MAIT cell numbers were linked to higher glucose concentrations which caused MAIT cell apoptosis. This study proposes circulating cell frequency as a marker for CMD progression (58). On the contrary, inhibition of MAIT cells was found to be neuroprotective in the model of ischemic brain injury as MAIT cell cytokines were found to cause neuroinflammation following ischemic reperfusion (67).

## Conclusions

A common feature in the metabolic diseases discussed, is that MAIT cells are altered in their frequency and function. In most of the metabolic diseases discussed, MAIT cells are implicated in pathogenesis, but in other such as T1DM, data from murine models suggest they have potential to be protective. As we expand our current understanding of MAIT cell biology, including their metabolic requirements, in both the steady state and diseased settings it is possible that we may be able to target MAIT cells; by either to inhibiting or restoring their functions. With cellular metabolism playing such a central role in MAIT cell biology and in particular their effector functionality, it warrants investigations into whether we can target MAIT cell metabolism to restore their protective functions in disease settings such as cancer or obesity. For example, we have shown that increased IL-17 production seen in obesity is a result of mitochondrial dysfunction in MAIT cells that increases ROS accumulation. Using ROS scavengers and antioxidants such as glutathione could improve MAIT cell health in obesity. Additionally, glutathione which controls ROS levels in cells has been shown to be instrumental for T cell metabolic reprogramming (79). In summary, it is very clear that MAIT cells are dysregulated in the setting of metabolic disease, and most-likely contributing to the pathogenesis, however the exact mechanisms underpinning these dysregulations are unclear. As the metabolic and nutritional requirements of MAIT cell emerge, it will be important to investigate if these are altered in disease settings and linked to MAIT cell dysregulation.

## Author contributions

NK-M & AH - performed the literature search, wrote, edited and approved the review. Both authors contributed to the article and approved the submitted version.
